# Surgical management for displaced pediatric proximal humeral fractures: a cost analysis

**DOI:** 10.1007/s11832-015-0643-2

**Published:** 2015-02-20

**Authors:** Benjamin J. Shore, Daniel J. Hedequist, Patricia E. Miller, Peter M. Waters, Donald S. Bae

**Affiliations:** Department of Orthopaedic Surgery, Boston Children’s Hospital, Harvard Medical School, Hunnewell 221, 300 Longwood Avenue, Boston, MA 02115 USA

**Keywords:** Pediatric proximal humerus fractures, Decision analysis, Cost analysis

## Abstract

**Purpose:**

The purpose of this investigation was to determine which of the following methods of fixation, percutaneous pinning (PP) or intramedullary nailing (IMN), was more cost-effective in the treatment of displaced pediatric proximal humeral fractures (PPHF).

**Methods:**

A retrospective cohort of surgically treated PPHF over a 12-year period at a single institution was performed. A decision analysis model was constructed to compare three surgical strategies: IMN versus percutaneous pinning leaving the pins exposed (PPE) versus leaving the pins buried (PPB). Finally, sensitivity analyses were performed, assessing the cost-effectiveness of each technique when infection rates and cost of deep infections were varied.

**Results:**

A total of 84 patients with displaced PPHF underwent surgical stabilization. A total of 35 cases were treated with IMN, 32 with PPE, and 17 with PPB. The age, sex, and preoperative fracture angulation were similar across all groups. A greater percentage of open reduction was seen in the IMN and PPB groups (*p* = 0.03), while a higher proportion of physeal injury was seen in the PPE group (*p* = 0.02). Surgical time and estimated blood loss was higher in the IMN group (*p* < 0.001 and *p* = 0.01, respectively). The decision analysis revealed that the PPE technique resulted in an average cost saving of $4,502 per patient compared to IMN and $2,066 compared to PPB. This strategy remained cost-effective even when the complication rates with exposed implants approached 55 %.

**Conclusions:**

Leaving pins exposed after surgical fixation of PPHF is more cost-effective than either burying pins or using intramedullary fixation.

## Introduction

Proximal humeral fractures in children represent about 2 % of all pediatric fractures [1], with a peak incidence between 11 and 15 years of age [2]. Purely epiphyseal proximal humeral injuries occur even more infrequently, with approximately 2.2–4.5 per 1,000 epiphyseal injuries per year [3, 4]. The physis of the proximal humerus accounts for 80 % of longitudinal growth of the upper arm and, hence, represents an enormous potential for the correction of residual axial deformities [1, 2, 5–8]. However, in older children with less growth remaining, severely displaced fractures may need operative treatment to restore anatomic alignment and maximize shoulder motion [5, 6, 9–11].

A variety of stabilization techniques have been described for the surgical management of pediatric proximal humerus fractures, including Kirschner wires [6, 9, 12–15], screws [9], and intramedullary nails [1, 6, 10, 16–18]. Despite preliminary reports on the use of percutaneous pins and intramedullary nails for pediatric proximal humeral fractures, a paucity of literature exists comparing the safety and efficacy of the two treatment techniques [9, 10, 13, 19]. Recently, at our institution, Hutchinson et al. [6] compared the results of percutaneous pinning (PP) versus retrograde intramedullary nailing (IMN) for proximal humeral fractures in the pediatric population. The authors concluded that both IMN and PP techniques have comparable short-term radiographic results; however, PP techniques have higher rates of pin-related complications, while IMN techniques generally require longer surgeries, greater blood loss, and higher rates of surgical implant removal.

The primary objective of this study was to determine which surgical strategy [percutaneous pinning leaving pins exposed (PPE) vs. percutaneous pinning leaving pins buried (PPB) vs. retrograde intramedullary nailing (IMN)] is most cost-effective for the treatment of displaced proximal humeral fractures. Data were obtained from both a retrospective study and a systematic review of the literature, and a decision analysis model was created to compare these three treatment strategies.

## Methods

### Retrospective review

After institutional review board approval, a retrospective review was performed of 107 displaced proximal humeral fractures, which underwent surgical reduction and PP (PPE or PPB) or IMN between 2000 and 2012. This review was an extension of the work done by Hutchinson et al. [6], which examined 56 children with displaced proximal humeral fractures between 2000 and 2009. The average age at injury was 13.8 years (range 8–17) and the average length of follow-up was 6 months.

Inclusion criteria included: (1) skeletal immaturity as determined by the presence of open physes and (2) displaced proximal humeral physeal or metaphyseal fractures deemed to be in unacceptable alignment given the patient age and remodeling potential. Surgical treatment involving open reduction (OR) or closed reduction (CR) and internal fixation using PPE, PPB, or IMN was recorded. Pre- and postoperative radiographic data were available for 84 patients: 35 treated with IMN, 32 treated with PPE, and 17 treated with PPB. The outcomes of interest included patient and injury demographics (age, gender, mechanism of injury, fracture type), surgical treatment characteristics (fixation type, surgical time, and estimated blood loss), and type of complications (superficial and deep infections, implant-related complications, and need for secondary surgery). A superficial infection was defined as local cellulitis with or without serous discharge treated with oral antibiotics. Any infection that warranted operative debridement was considered a deep infection. Radiographs were assessed for maximum angular deformity and Neer–Horowitz classification on preoperative, immediate postoperative, and final follow-up plain films [2, 6].

All patients were treated by 15 fellowship-trained, pediatric orthopedic surgeons at a tertiary care pediatric hospital. General indications for surgical treatment were patients aged 12 or more years with Neer–Horowitz grade four fractures or angulation of 40° or more; however, the treating attending surgeon made the ultimate determination for surgical intervention. Patients were taken to the operating room for attempted CR and fixation with either PP (buried or exposed) or IMN. If CR could not be obtained, OR using a deltopectoral approach was performed to facilitate reduction prior to internal fixation. All patients received preoperative antibiotics between 30 and 60 min prior to procedure and a total of 24 h of antibiotics was administered for those patients who underwent open reduction and fixation. For PP fixation, 2 or 3 pins were placed [average 2.4 pins (5/64 or 3/32 inch diameter)]. Pins were placed through the lateral metaphysis of the distal fracture fragment and passed superomedially across the fracture site into the humeral head fragment. Pins were started inferiorly and only after careful blunt spreading of the subcutaneous tissues so as to avoid iatrogenic injury to the axillary nerve. In rare situations, a third pin was started in the greater tuberosity and passed inferomedially across the fracture site and into the medial humeral cortex of the distal fragment. Pins were cut beneath the skin in 35 % (17/49) of cases. The decision to bury pins or leave them through the skin was based on surgeon preference. IMN fixation was employed with 1 or 2 titanium flexible nails (Synthes, West Chester, PA), through a distal lateral entry site. Nails were introduced by making a small incision and spreading bluntly down to bone, where a drill was used to make an entry portal into the intramedullary canal superior to the olecranon fossa. The nails were then driven retrograde until they entered the proximal fragment. If necessary, rotation of the nail was used to optimize reduction. IMN implants were then trimmed and buried under the skin in all cases. In all cases, wounds were dressed with antibiotic impregnated gauze, dry dressing, and sterile silk tape, which remained in place until the first postoperative visit. In general, a sling and swathe was used for postoperative immobilization.

### Decision analysis model

**Fig. 1:**
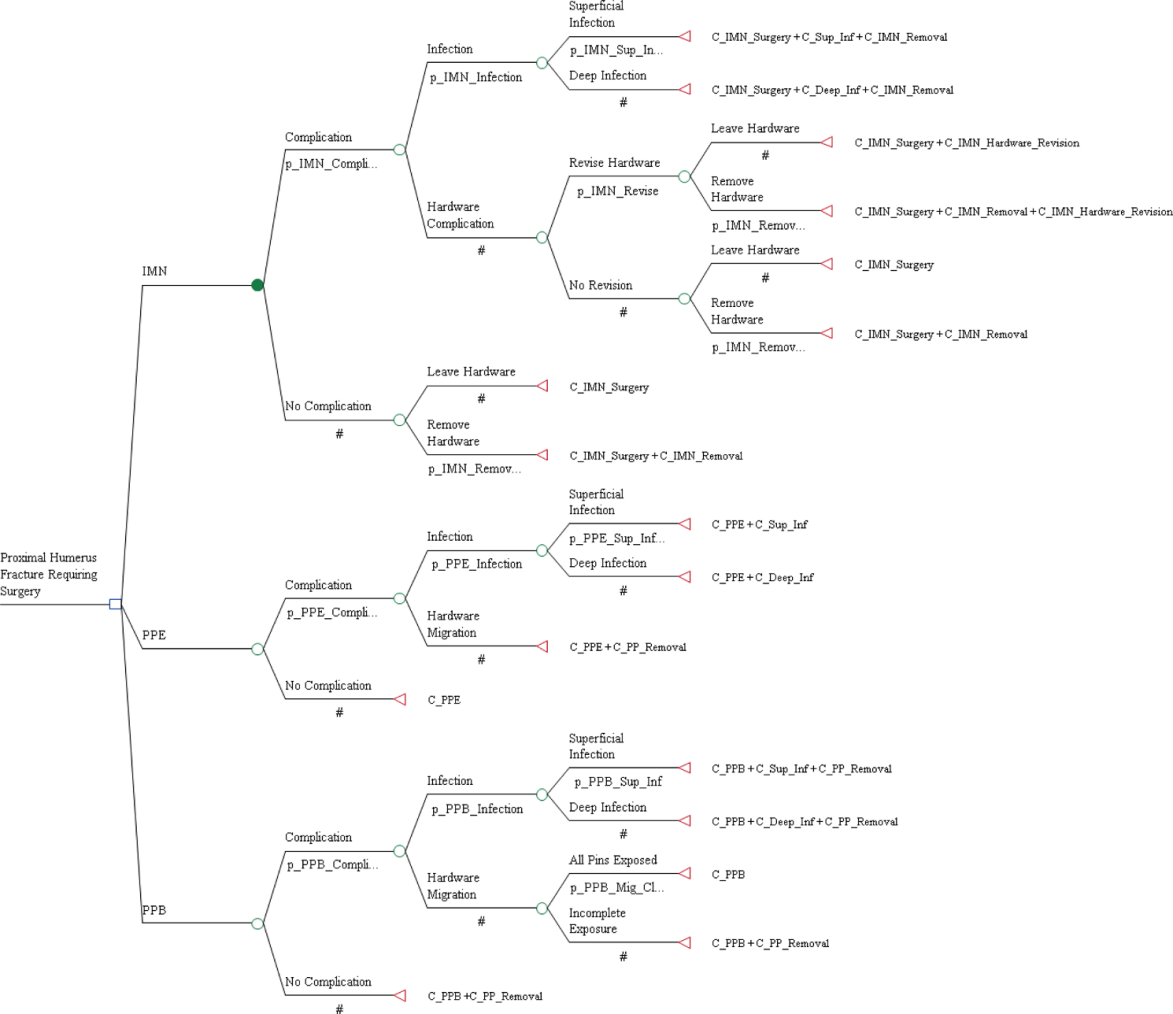
Decision tree used to compare the three treatment techniques for pediatric proximal humerus fractures: intramedullary nailing (IMN), exposed percutaneous pinning (PPE), and buried percutaneous pinning (PPB). Symbols: *square* decision node; *circle* chance node; *triangle* outcome/terminal node. Numerical values represent outcome probabilities. *#* represents the complement probability such that the sum of probabilities at a node is 1.00 (e.g., if p_IMN_Complication = 0.35, then # takes a value of 0.65). For example: p_IMN_Complication: probability of having a complication after IMN insertion, including infection (superficial vs. deep) or hardware complication (migration/protrusion). C_PPE + C_Sup_Inf: cost of exposed percutaneous pin insertion and cost of superficial skin infection. A list of all probabilities and costs are included as follows: *probabilities*: p_IMN_Complication = probability of IMN complication; p_IMN_Infection = probability of infection with IMN nailing (_Sup = superficial infection); p_IMN_Revise = probability of hardware complications with IMN; p_IMN_Removal = probability of removing hardware after IMN; p_PPE_Complication = probability of PPE complication; p_PPE_Infection = probability of infection with PPE (_Sup = superficial infection); p_PPB_Complication = probability of PPB complication; p_PPB_Infection = probability of infection with PPB (_Sup = superficial infection); p_PPB_Mig_Cl = probability of all hardware migration with PPB removed in clinic; *cost*: C_IMN = cost of IMN insertion; C_PPE = cost of PPE; C_PPB = cost of PPB; C_IMN_ + C_Sup_Inf + C_IMN_Removal = cost of IMN insertion, superficial infection, IMN removal; C_IMN_ + C_Deep_Inf + C_IMN_Removal = cost of IMN insertion, deep infection, IMN removal; C_IMN_ + C_IMN_Removal = cost of IMN insertion and removal; C_IMN_ + C_IMN_Removal + C_IMN_Hardware_Revision = cost of IMN insertion, revision, and removal; C_PPE + C_Sup_Inf = cost of PPE and cost of superficial infection; C_PPE + C_Deep_Inf = cost of PPE and cost of deep infection; C_PPE + C_PP_removal = cost of PPE and hardware removal; C_PPB + C_PP_removal = cost of PPB and hardware removal; C_PPB + C_Sup_Inf + C_PP_removal = cost of PPB, superficial infection, and pin removal; C_PPB + C_Deep_Inf + C_PP_removal = cost of PPB, deep infection, and pin removal

To compare the three strategies of surgical treatment for displaced proximal humeral fractures (PPE vs. PPB vs. IMN], a cost analysis decision model was constructed. Several assumptions which vary from daily practice were required for this model to function accurately: (1) each patient could have only one complication; (2) only complications related to the fixation technique were included in the model; any complication related to the surgical approach or fracture type was omitted, as they were assumed to occur with equal likelihood in either treatment group; (3) any complication would completely resolve after treatment was instituted; and (4) the technique of humeral fixation was unlikely to have long-term consequences on patient outcome (Fig. 1) [6].

In accordance with the recommendations of the Panel on Cost-Effectiveness in Health and Medicine [20], a societal perspective was adopted, which takes into account the cost of all resources consumed, regardless of who actually experiences them. Sensitivity analysis was performed to investigate the impact of parameters that were either difficult to estimate with certainty or where considerable variation existed from our retrospective analysis and review of the literature [21].

### Outcome probabilities

A literature review was performed to identify outcomes and complications associated with various fixation methods for displaced proximal humeral fractures [1–3, 5–13, 17–19, 22–26]. Complications that were likely to affect the treatment costs included: superficial and deep infection, pin protrusion, and pin migration requiring repeat surgery. Minor complications, which did not have a measurable effect on overall treatment costs, were omitted (hypertrophic scar, sensitivity over incision, or bursitis).

A decision tree outlining the three surgical scenarios was constructed with one decision branch (decision node), 14 chance branches (chance nodes), and 17 outcomes (terminal nodes) (Fig. 1). In the IMN scenario, implant-related complication refers to migration of wires or loss of fixation requiring revision surgery. In the PPE scenario, implant migration refers to one or several pins migrating under the skin requiring a second operation to remove. Finally, in the PPB scenario, implant migration refers to where buried pins protrude through the skin. If one or more pins were still buried, a second operation for implant removal was still required.

### Sensitivity analysis

A decision analysis model is based several assumptions. In this scenario, many of the outcome estimates and probabilities have a wide range of values. Sensitivity analysis is a technique used to help examine this uncertainty [21]. One-way sensitivity analysis was performed to model the effect of varying the infection rate (in exposed pins) and the cost of treating deep infections. In our review, the overall rate of infection from PPE ranged from 0 to 57 % [2, 5–7, 9, 12, 13, 19, 20, 27] and it was assumed that the proportion of superficial infection from this cohort varied from 30 to 100 %. The treatment of deep infection encompassed a broad spectrum of severity, ranging from a single irrigation in the operating room, to repeated procedures, negative pressure dressings, and long-term antibiotics. To account for this variation in the cost of treatment for deep infection, we used a similar technique as previously described, using a lower limit of 20 % below the lowest cost estimate and a 50 % estimate above the highest cost estimate [28].

### Costs

Total treatment costs were estimated for each surgical scenario within the decision tree. For each scenario, we considered the charges of the implant, hospital admission, surgeon and anesthesia fees, operating room costs, nursing fees, admission fees, diagnostic imaging, medication, and amount of work productivity lost for one parent during the convalescent period of the child. Accurate costs were obtained from the appropriate billing departments within the hospital. Parental productivity loss during the period of their child’s injury was estimated from the population census data published by the U.S. Census Bureau [29].

For an uncomplicated case, we assumed either parent would lose 1 week of work during the entire period from initial injury to surgery and subsequent outpatient treatment and rehabilitation. For a superficial infection, we assumed either parent would lose 10 days of work during the entire period from initial injury to surgery and subsequent outpatient treatment and rehabilitation. In the setting of deep infection, we assumed that there would be additional time lost from work; this variation was captured in the sensitivity analysis [28]. Costs common to all three scenarios were omitted from the decision analysis model.

### Statistical analysis

A variety of statistical analyses using SAS (SAS Institute Inc., Cary, NC) were performed. Analysis of variance (ANOVA) was employed for continuous variables and Tukey’s multiple comparisons was used for post hoc comparisons. Estimated blood loss was compared using a Kruskal–Wallis test and Chi-square testing was used for binary and categorical variables. *p*-Values of less than 0.05 were considered statistically significant. Outcome probabilities and costs were analyzed using TreeAge software (TreeAge Software Inc., Williamstown, MA).

## Results

### Retrospective review

**Fig. 2:**
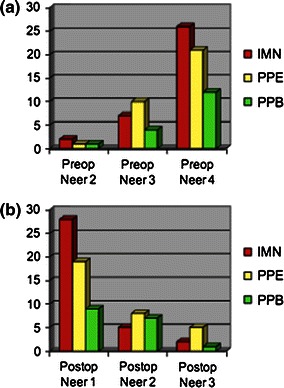
Preoperative and postoperative Neer–Horowitz classifications for the three treatment groups: intramedullary nailing (*IMN*), exposed percutaneous pinning (*PPE*), and buried percutaneous pinning (*PPB*). No statistically significant changes were seen in the proportions of fracture type across each of the three groups

A total of 84 patients were included in our retrospective analysis (age 13.8 ± 2.25 years); 35 cases were treated with IMN, 32 cases with PPE, and 17 cases with PPB. There were no differences in age, gender, Neer–Horowitz classification, and pre/postoperative angulations across the three surgical groups (Fig. 2a, b). A greater proportion of open reductions was seen in the IMN and PPB groups compared to the PPE group (*p* = 0.03), while a higher proportion of physeal fractures was seen in the PPE group (*p* = 0.02) (Table 1). A fall or sports-related injury was the most common injury mechanism across all three groups. All patients achieved significant improvements in angulation and Neer–Horowitz score on the final radiograph. The average preoperative angulation was 44.2° compared with 12.4° on the final radiograph (*p* < 0.001), but there was no significant difference in angulation amongst treatment groups, nor was there a significant difference in the change of angulation between immediate postoperative and final postoperative radiographs between groups (Table 1). All patients went on to achieve clinical and radiographic healing without functionally limiting loss of global shoulder motion compared with contralateral shoulder, pain, or weakness.

**Table 1:** Comparison of intramedullary nailing versus percutaneous pinning groups

	Total, *n* = 84	PPE, *n* = 32	PPB, *n* = 17	IMN, *n* = 35	*p*-value
Age, mean ± SD	13.8 (± 2.25)	13.8 (± 2.67)	14.0 (± 1.29)	13.6 (± 2.24)	0.81
Sex (male)	61 (73 %)	26 (81 %)	13 (76 %)	22 (63 %)	0.22
Reduction (open)	36 (43 %)	8 (25 %)	9 (53 %)	19 (54 %)	0.03
Location (physeal)	48(57 %)	24 (75 %)	10 (59 %)	14 (40 %)	0.02
MOI					
Fall	18 (21 %)	3 (9 %)	2 (12 %)	13 (37 %)	0.04
Sports	53 (63 %)	24 (75 %)	13 (76 %)	16 (46 %)	
MVA	7 (8 %)	4 (13 %)	0 (0 %)	3 (9 %)	
Other	6 (7 %)	1(3 %)	2 (12 %)	3 (9 %)	
EBL, median (IQR)	20 (2–150)	5 (0–40)	20 (0–100)	40 (6–238)	0.01
Surgical time, mean ± SD	87.4 (± 52.8)	64.2 (± 35.97)	74.4 (± 43.05)	115 (± 58.15)	<0.001
Max preoperative angulation, mean ± SD	44.2 (± 17.3)	47.6 (± 16.7)	48.9 (± 18.1)	39.5 (± 16.9)	0.13
Max postoperative angulation, mean ± SD	14.6 (± 12.5)	18.8 (± 15.9)	13.4 (± 8.5)	11.4 (± 9.2)	0.06
Max final angulation, mean ± SD	12.4 (± 10.1)	13.4 (± 10.9)	16 (± 12.2)	9.4 (± 7.1)	0.07

*MOI* mechanism of injury, *MVA* motor vehicle accident, *EBL* estimated blood loss, *IQR* interquartile range, *SD* standard deviation, *Max* maximum, *PPE* percutaneous pins exposed, *PPB* percutaneous pins buried, *IMN* intramedullary nailing

The overall median estimated blood loss was 20 cc [interquartile range (IQR) 2–150]; however, patients undergoing IMN lost a median of 40 cc (IQR 6–238) compared to a median of 20 cc (IQR 0–100) for PPB and 5 cc (IQR0–40) for PPE (*p* = 0.01). While the differences above are statistically significant, there is no clinically significant difference between a 40 and 5 cc blood loss and, thus, from a clinical perspective, blood loss was equivocal between treatment groups. The overall mean surgical time was 87.4 min (± 52.8); however, for patients undergoing IMN, the surgical time was 115 min (± 58.15) compared to 74.4 min (± 43.05) for PPB and 64.2 min (± 35.97) for PPE (*p* < 0.001) (Table 2).

**Table 2:** Post hoc comparisons of continuous variables

Variable	IMN vs. PPB	IMN vs. PPE	PPB vs. PPE
Age	0.80	0.97	0.90
Surgical time	0.01	<0.001	0.83
EBL	0.06	0.004	0.90
Max preoperative angulation	0.30	0.17	0.99
Max postoperative angulation	0.86	0.06	0.39
Max final angulation	0.08	0.34	0.57

*EBL* estimated blood loss, *Max* maximum, *IMN* intramedullary nailing, *PPB* percutaneous pin buried, *PPE* percutaneous pin exposed

A summary of complications is seen in Table 3. The overall complication rate was significantly higher in the PPE and PPB groups (both 41 %) compared to the IMN group (11 %) (*p* = 0.01) (Table 3). A greater percentage of implant-related complications was seen in the PP groups, with four in the PPB group (24 %) and seven in the PPE group (22 %), compared to only two in the IMN group (6 %), but this difference was not significant (*p* = 0.11). Although a three times higher rate of wound infection was seen with the PP techniques, this was not statistically significant (*p* = 0.24). Thirty-one patients with IMN (89 %) underwent repeat surgery for implant removal and two had implant-related complications related to prominent nails at the elbow. There were seven patients who underwent PPE (22 %) who required a second operation as a result of pin migration under the skin. Four patients suffered implant complications in the PPB group; one patient had all three exposed pins removed in the clinic, while the remaining three required a secondary operation.

**Table 3:** Comparison of complications by surgical technique

Type of complication	IMN (%)	PPB (%)	PPE (%)
Superficial infection	1 (0.03)	2 (0.12)	4 (0.13)
Deep infection	1 (0.03)	1 (0.06)	2 (0.06)
Hardware migration	2 (0.06)	4 (0.24)	7 (0.22)
No complications	31 (0.87)	10 (0.59)	19 (0.59)

Total complications	No.	Frequency	*p* value
IMN	35	4 (0.11)	0.01
PPB	17	7 (0.41)	
PPE	32	13 (0.41)	

Any infection	No.	Frequency	*p* value
IMN	35	2 (0.06)	0.24
PPB	17	3 (0.18)	
PPE	32	6 (0.19)	

Hardware migration	No.	Frequency	*p* value
IMN	35	2 (0.06)	0.11
PPB	17	4 (0.248)	
PPE	32	7 (0.22)	

*IMN* intramedullary nailing, *PPB* percutaneous pins buried, *PPE* percutaneous pins exposed

### Outcome probabilities

The outcome probabilities that were used in the decision model and range of sensitivity analysis are shown in Table 4. A wide range of infection rates after surgical treatment of proximal humerus fractures has been previously reported. Yet, to our knowledge, only one previous study has compared all three surgical techniques and their associated complication rates [6].

**Table 4:** Outcome probabilities used in the decision analysis model

Event	Estimate used in the decision model (%)	Probability range in the literature	Range used for sensitivity analysis (%)
PPE			
Total complication rate	41	0–57 [6, 9, 12, 19, 20, 24]	0–80
Total infection rate	19	0–17 [6, 9, 12, 19, 20, 24]	30–100
Proportion of infections that were superficial	67	80–100 [6, 12, 19]	
Hardware complication rate	22	0–48 [12, 19]	
PPB			
Total complication rate	41	0–57 [6, 9, 12, 19, 20, 24]	
Total infection rate	18	0–17[6, 9, 12, 19, 20, 24]	
Proportion of infections that were superficial	67	80–100 [6, 12, 19]	
Hardware complication rate	24	0–48 [12, 19]	
IMN			
Total complication rate	11	0–25 [1, 6, 10, 17, 18, 23]	
Total infection rate	6	0–4 [1, 6, 10, 17]	
Proportion of infections that were superficial	50	67–100 [6, 10, 18]	
Hardware complication rate	6	0–21 [6, 10, 17, 18]	

*IMN* intramedullary nailing, *PPB* percutaneous pins buried, *PPE* percutaneous pins exposed

The mean costs for each treatment scenario is shown in Table 5. There were only four cases of deep infection within this cohort and, as a result, the range of treatment costs are shown. The treatment costs for deep infection used in the sensitivity analysis ranged from $4,631 to $40,245.

**Table 5:** Cost estimates for each outcome scenario

Scenario	Average cost ($)	Range used for sensitivity analysis
Cost of IMN insertion (*n* = 35)		
OR/anesthesia/RR	6,323	
Diagnostic services	713	
Emergency services	199	
General nursing	3,234	
Pharmacy	830	
Other	179	
Total	11,478	
Cost of PP insertion (*n* = 52)		
OR/anesthesia/RR	5,043	
Diagnostic services	451	
Emergency services	443	
General nursing	2,157	
Pharmacy	463	
Other	148	
Total	8,705	
Cost of hardware removal (*n* = 54)		
OR/anesthesia/RR	2,794	
Diagnostic services	63	
Emergency services	0	
General nursing	0	
Pharmacy	57	
Other	27	
Total	2,941	
Cost of deep infection (*n* = 4)		
OR/anesthesia/RR	6,380	
Diagnostic services	1,426	
Emergency services	250	
General nursing	3,285	
Pharmacy	830	
Other	95	
Total	12,121 (range 5,789–26,830)	$4,631–$40,245
Cost of superficial infection (*n* = 7) (oral cephalexin for 10 days)	15	
Average weekly US salary	771	

*OR* operating room, *RR* recovery room, *IMN* intramedullary nail, *PP* percutaneous pin

### Decision analysis

**Fig. 3:**
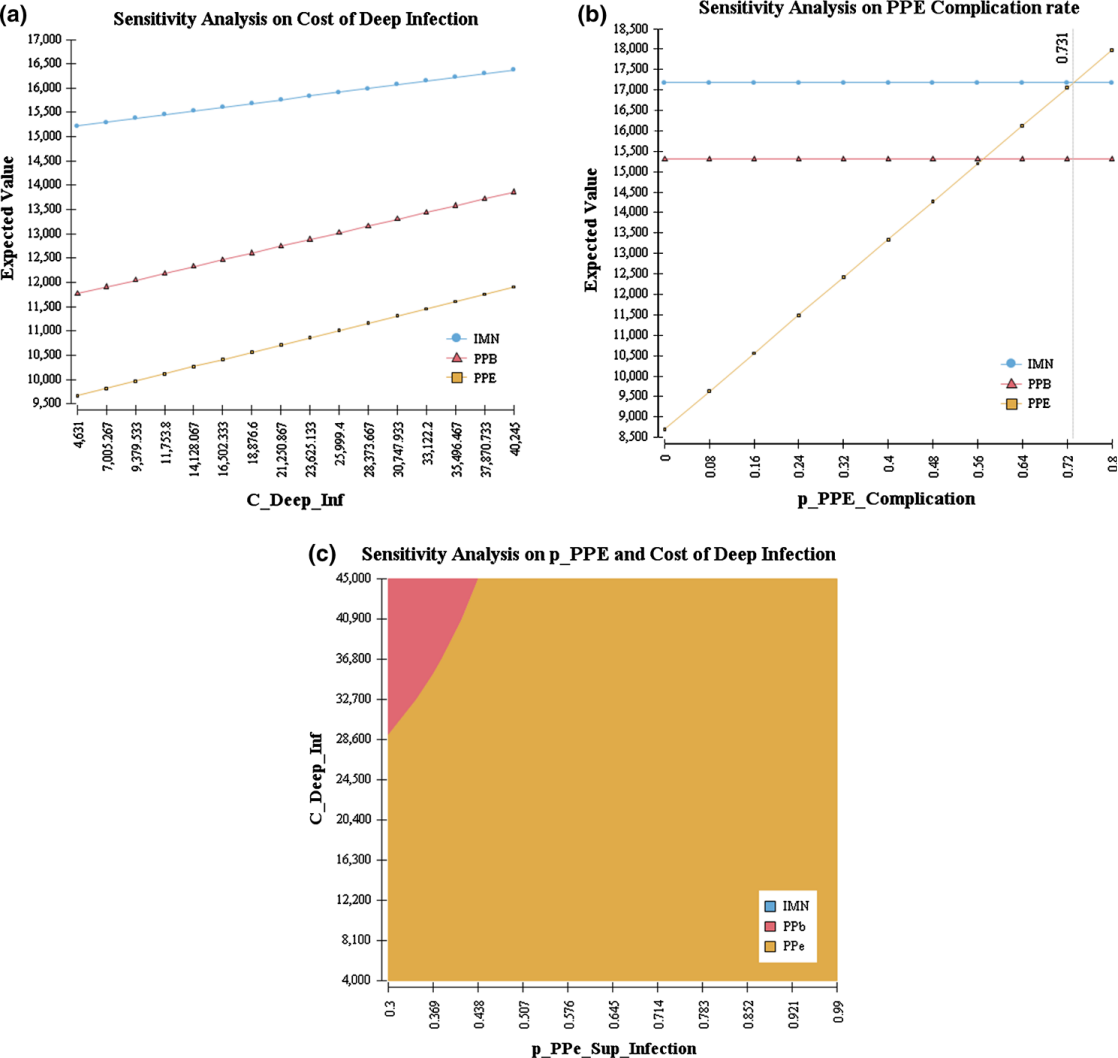
One-way and two-way sensitivity analyses. **a** One-way sensitivity analysis on cost of deep infection: cost of treating deep infection varied from $4,631 to $40,245. The “Expected Value” refers to the mean treatment cost per patient using a particular strategy. This analysis shows that leaving the pins exposed (*squares*) consistently results in lower treatment costs than burying the pins (*triangles*) or intramedullary nailing (*circles*). **b** One-way sensitivity analysis on PPE complication rate: the PPE complication rate varied from 0 to 0.8. The “Expected Value” refers to the mean treatment cost per patient using a particular strategy as the PPE complication rate varied. Note that the “Expected Value” for the PPB (*triangles*) and IMN (*circles*) strategies does not change but, as the PPE complication rate rises above 0.56, the PPB strategy becomes most cost-effective, and when the PPE complication rate rises above 0.72, the IMN strategy is more cost-effective than the PPE (*squares*) strategy. **c** Two-way sensitivity analysis: the proportion of all infections that were superficial (*x*-axis) varied from 30 to 100 % and the cost of treating deep infection (*y*-axis) varied from $4,000 to $45,000. Leaving the pins exposed (*yellow*) results in greater cost savings than leaving them buried (*pink*), except when the cost of infection rises above $28,600 and the rate of superficial infection is low, between 0.3 and 0.438. Under no circumstances was the IMN strategy cost-effective through the modeled cost and outcome probabilities

The decision analysis revealed that leaving pins exposed (PPE) after operative fixation of proximal humerus fractures was the most cost-effective strategy. Specifically, a per patient average cost saving of $4,502 was seen compared to the IMN strategy and $2,066 compared to the PPB strategy. The one-way sensitivity analysis demonstrated that PPE was cost-effective through a wide range of costs for treating deep infection (Fig. 3a). This strategy remained cost-effective when the complication rates associated with exposed implants approached 55 % (Fig. 3b). The two-way sensitivity analysis demonstrated that the PPE scenario remained the most cost-effective strategy across a variable rate of superficial infection and cost of deep infection (Fig. 3c).

## Discussion

Although most pediatric proximal humeral fractures can be successfully treated non-operatively, multiple authors recommend surgical stabilization in older patients with highly displaced fractures [6, 9, 22, 24]. The results of this study demonstrate that leaving pins exposed after surgical treatment of pediatric proximal humeral fractures is safe and confers greater cost savings than burying the pins or using intramedullary fixation. Our sensitivity analysis demonstrated that these findings remained true despite employing a wide range of probable infection rates and treatment costs.

There are several safe and effective surgical techniques to manage pediatric proximal humerus fractures. Similar to Hutchinson et al. [6], both IMN and PP fixation (PPE and PPB) provided adequate stability and maintenance of reduction in the immediate postoperative period for both physeal and metaphyseal fractures. Given that all three (IMN, PPE, and PPB) techniques were effective in the treatment of proximal humeral fractures, the choice of technique should be made based on the advantages and disadvantages of each strategy. A higher proportion of open reductions was seen in the IMN and PPB groups, where the majority of fractures where metaphyseal rather than physeal, illustrating the fact that many of these injuries had interposed soft tissue blocking the reduction. In our study, we saw that IMN had fewer complications but longer surgeries, higher blood loss, and an almost 90 % rate of secondary surgery for implant removal. Conversely, PPE had shorter surgeries and lower estimated blood loss (EBL) but more superficial skin infections and hardware complications. A greater proportion of physeal injuries were treated with PPE, demonstrating the belief that physeal injuries heal quickly, allowing for exposed hardware and early removal compared to more distal metaphyseal injuries. Finally, children treated with PPB experienced a 100 % rate of secondary surgery for hardware removal and a lower complication rate than PPE with similar blood loss and surgical time. Ultimately, the final treatment decision was made at the discretion of the treating physician.

One of the main concerns associated with leaving implants exposed is the increased risk of infection. For many pediatric surgeons, the rationale for leaving pins buried is a perceived lower risk of infection; in contrast, surgeons who elect to leave pins exposed did so to facilitate easier removal and avoidance of a costly secondary surgery. Our review of the literature demonstrated a wide variation in the rates of infection after proximal humerus fractures (0–17 % [6, 9, 12, 19, 20, 24]). Within our retrospective review, we were unable to identify a significant difference in the rate of infection between the three treatment strategies; however, patients treated with IMN had a three times lower rate of infection compared to the two PP scenarios. Pin migration associated with PPE was increased in our cohort at 25 %; to help mitigate this risk, we have moved to a more aggressive immobilization strategy after surgery including a sling and swathe to limit shoulder motion, as we believe that this motion creates tension on the exposed wires, leading to pin migration.

The results of the decision analysis demonstrate that the main factor affecting the cost of treatment was the requirement for a secondary surgery for implant removal (approximately $3,000 in this cohort). As a result, the PPE scenario was the most cost-effective as long as the proportion of PPE infections remained primarily superficial. Despite the notion that PPE leaves pins exposed to obviate the need for a secondary surgery, almost 25 % of the PPE cohort experienced pin migration and required a repeat operation for implant removal. Other cost-effective options for pin removal include utilization of a clinical procedure room to facilitate removal under light sedation or with the adjunct of local anesthetic.

The advantage of using a decision analysis model is the ability to combine complex information on outcomes, complications, and costs, particularly in situations where uncertainty exists [30]. However, decision analysis is not without limitations; for our model (Fig. 1) to function, we assumed that each patient would have only one complication. We recognize that this is not accurate in real life; however, in order to develop a working model for displaced pediatric proximal humeral fractures, this assumption was necessary. Furthermore, cost analysis, when performed in this manner, is also limited, as costs and practices vary significantly across countries and continents. Despite these limitations, we believe that important lessons can be gleaned from this model and analysis.

This study is further limited by its retrospective design, selection bias, and surgeon experience/performance bias. While age, gender, Neer–Horowitz classification, and preoperative angulation were similar across all treatment groups, surgeon familiarity and experience may ultimately be the determining factors in deciding which treatment scenario is chosen. Furthermore, we have no information on patients' or parents' utilities according to each complication. While the PPE scenario is the most cost beneficial, if we took into account patient and parental values regarding repeat surgical intervention and antibiotic administration, the most desirable scenario most likely would have changed. While this study was appropriately powered to answer the question of cost-effectiveness, it was likely underpowered to detect clinically important differences across treatment groups. The small sample sizes in each of the three scenarios further limited our analysis. We observed statistically significant differences in EBL and surgical time, which likely had little clinically significant effects on the overall outcome, and these results need to be interpreted cautiously. Finally, our analysis is limited by the relatively short-term follow-up of our cohort and, as a result, we are unable to make definitive conclusions on the long-term functional differences between each treatment scenario.

In conclusion, leaving pins exposed after surgical fixation of pediatric proximal humeral fractures is safe and more cost-effective than either burying pins or using intramedullary fixation. Employing the exposed pin strategy has the potential to provide a cost saving of approximately $4,500 per patient treated.
